# Dual Therapeutic Action of a Neutralizing Anti-FGF2 Aptamer in Bone Disease and Bone Cancer Pain

**DOI:** 10.1038/mt.2016.158

**Published:** 2016-09-27

**Authors:** Ling Jin, Yosuke Nonaka, Shin Miyakawa, Masatoshi Fujiwara, Yoshikazu Nakamura

**Affiliations:** 1 Ribomic Inc., Tokyo, Japan; 2 Institute of Medical Science, The University of Tokyo, Tokyo, Japan

## Abstract

Fibroblast growth factor 2 (FGF2) plays a crucial role in bone remodeling and disease progression. However, the potential of FGF2 antagonists for treatment of patients with bone diseases has not yet been explored. Therefore, we generated a novel RNA aptamer, APT-F2, specific for human FGF2 and characterized its properties *in vitro* and i*n vivo*. APT-F2 blocked binding of FGF2 to each of its four cellular receptors, inhibited FGF2-induced downstream signaling and cells proliferation, and restored osteoblast differentiation blocked by FGF2. APT-F2P, a PEGylated form of APT-F2, effectively blocked the bone disruption in mouse and rat models of arthritis and osteoporosis. Treatment with APT-F2P also exerted a strong analgesic effect, equivalent to morphine, in a mouse model of bone cancer pain. These findings demonstrated dual therapeutic action of APT-F2P in bone diseases and pain, providing a promising approach to the treatment of bone diseases.

## Introduction

The fibroblast growth factor (FGF) family plays important roles in cells proliferation, differentiation, and migration.^1,2^ The FGF family has 22 known members in humans, including FGF1 and FGF2 (also called acidic FGF and basic FGF, respectively). FGF1 and FGF2 are the most abundant in adult tissues. FGFs bind tyrosine-kinase receptors, FGF receptors 1–4 (designated FGFR1- FGFR4), activating various signaling pathways, in particular mitogen-activated protein kinase (MAPK), extracellular signal-regulated kinase (ERK), and JNK. In addition to binding all the receptors FGFR1–4 with high affinity, FGF2 binds to heparin sulfate proteoglycans with lower affinity.

Human FGF2 is an 18-kDa nonglycosylated polypeptide consisting of 146 amino acids in the mature form derived from a 155 amino acid precursor.^3^ Although the precursor does not encode a signal sequence, FGF2 is secreted during the healing process of fractures or in surgery bone sites^4^ by an unconventional pathway.^5^

Bone is a complex organ that has multiple functions in the body. It provides a structural framework, is a storehouse for calcium, and is the site of hematopoiesis. To maintain these functions, bone constantly remodels. Bone resorption is accomplished by osteoclasts, which remove bone, whereas osteoblasts are the cells that form bone.^6^ FGF2 is a potent stimulator of premature osteoblast proliferation,^7^ as well as a potent suppressor of osteoblast differentiation,^8,9,10^ is produced by osteoblasts^11,12^ and stored in the extracellular matrix,^13^ serving as an important regulator of bone and cartilage cells.^14^ Both osteoblasts and osteoclasts express the receptor for FGF2,^15^ thereby FGF2 modulates bone remodeling by interacting with, or coupling, both osteoblasts and osteoclasts. Disruption of FGF receptors is associated with severe skeletal defects,^16,17^ while targeted deletion of FGF2 in mice causes a relatively subtle defect in osteoblastogenesis, leading to decreased bone growth and bone density.^18^

FGF2 is involved in tissue remodeling and regeneration such as for skin wound healing, repair of neuronal damage, control of hypertension and in joint protection in osteoarthritis models. Although FGF2 might generally play a positive role in bone health, it exerts multiple actions on bone, depending on the developmental stage and disease conditions. In fact, several *in vitro* studies have suggested that FGF2 enhances bone disease progression^9,19,20,21^ (see **Supplementary Figure S1**). First, FGF2 inhibits the production of osteoprotegerin (OPG) by human fibroblast-like synovial (HFLS) cells.^22^ OPG is also called osteoclastogenesis inhibitory factor and represents a decoy receptor for the receptor activator of nuclear factor kappa B ligand (RANKL).^23^ OPG binding to RANKL on osteoblast cells, blocks the RANKL–RANK interaction between osteoblast cells and osteoclast precursors. This has the effect of inhibiting the differentiation of the osteoclast precursor into a mature osteoclast. FGF2 blocks the OPG production and reverses the osteoclast maturation. Second, FGF2 stimulates the production of RANKL in osteoblast cells.^24^ This has the effect of promoting the differentiation of the osteoclast precursor into a mature osteoclast. Third, FGF2 is a potent angiogenic factor.^25^ Inflammation and angiogenesis are tightly correlated in the pathophysiology of acute and chronic bone diseases such as rheumatoid arthritis (RA). Inflammation is normally a protective response to pathogenic, traumatic, or toxic injury, reducing tissue damage and leading to resolution or repair.^26^ In chronic inflammatory diseases such as RA, persistent inflammation itself causes tissue damage,^27^ leading to chronic pain and disability.^28,29^ It has been suggested that conversion of acute inflammation to chronic inflammation is due to the stimulation of angiogenesis, and brief antiangiogenic treatment during the acute phase of synovitis may prevent its subsequent progression.^30^ Collectively, the up-regulation of FGF2 seems to worsen bone diseases as a multiple-worsening factor (see **Supplementary Figure S1**). The *in vivo* role of FGF2 in bone diseases remains to be investigated.

Recent progress has been made in our understanding of how FGF2 signaling controls bone formation, and indicated that pharmaceutical manipulation of the FGF2 signaling pathway represents a promising approach to the treatment of bone diseases.

A number of anti-FGF2 monoclonal antibodies (mAbs) have previously been developed and shown to neutralize various activities of FGF2 *in vitro* and in some cases *in vivo*.^31,32,33,34,35^ However, to our knowledge, no anti-FGF2 mAb has been entered into clinical trials.

To firmly validate FGF2 as a therapeutic target and to initiate clinical testing, in this study, we developed and characterized an RNA aptamer to human FGF2. An aptamer is a short single-stranded nucleic acid molecule that is selected *in vitro* from a large random sequence library based on its high and specific affinity to a target molecule by a process known as Systematic Evolution of Ligands by EXponential enrichment (SELEX).^36,37^ Aptamers are applicable to therapeutics by strong and specific neutralizing activities, and hold several pharmaceutical advantages compared with antibodies such as a medium size between antibodies and small molecules, chemical synthesis, production cost, and little antigenicity.^38^

## Results

### Selection and binding properties of anti-FGF2 aptamer

**Figure 1:**
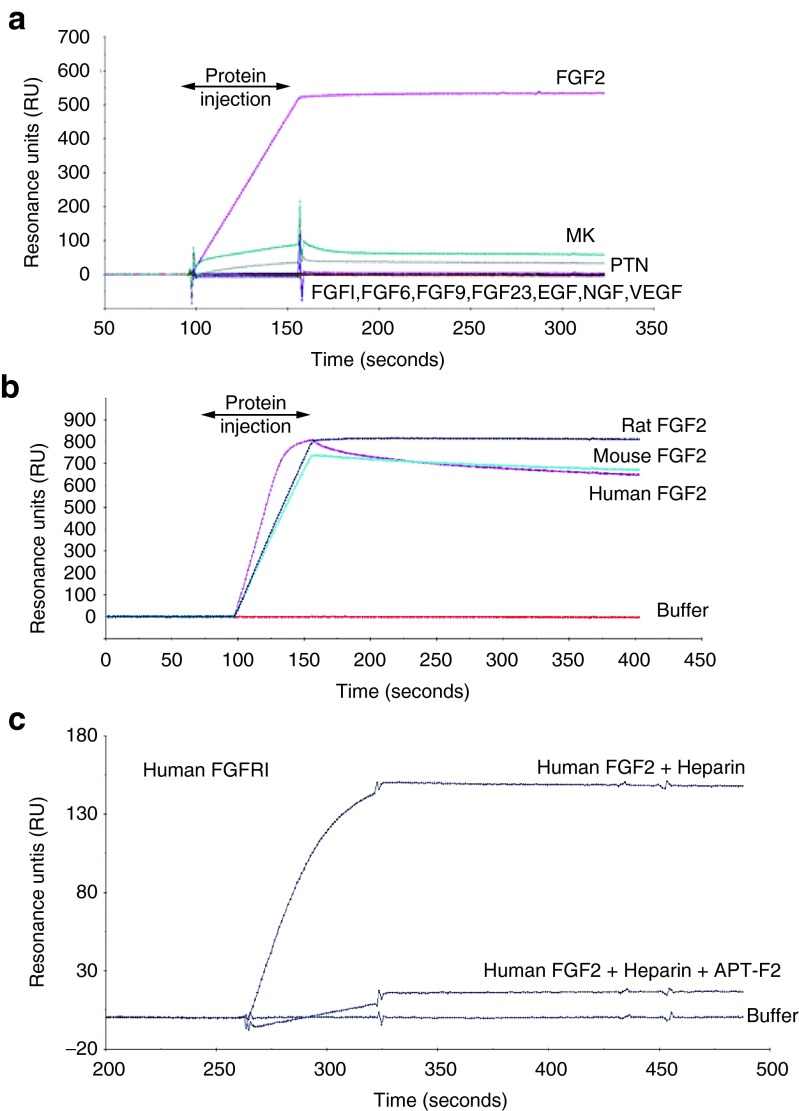
**Binding profiles of anti-FGF2 aptamer.** (**a**) SPR sensorgrams monitoring the affinity of APT-F2 to human FGF2 and other FGF family proteins or heparin-binding growth factors. The 3′-biotine labeled APT-F2 RNA was immobilized to a streptavidin sensor chip and the test proteins (100 nmol/l) were injected at the indicated time periods. Experimental conditions and procedures are described in Methods. (**b**) SPR sensorgrams showing the affinity of APT-F2 to the indicated different FGF2 species. (**c**) SPR sensorgrams showing the ability of APT-F2 to block the FGF2•FGFR1 interaction. Human FGFR1α(IIIc)/Fc fusion protein (100 nmol/l) was immobilized to a protein A sensor chip, and human FGF2 (100 nmol/l) was injected in the presence of heparin (100 nmol/l) with APT-F2 (150 nmol/l). FGF2, Fibroblast growth factor 2; SPR, surface plasmon resonance; FGFR, FGF receptor.

Aptamers were selected against human FGF2 mainly by the primer-less SELEX method^39^ from RNA pools randomized over 30 nucleotides (nt) with 2′-fluoro pyrimidine modifications to resist ribonucleases. Four hundred aptamers were obtained by different SELEX procedures and screened for the affinity to FGF2 and the inhibition of FGF2 binding to the FGFR1 receptor by a surface plasmon resonance (SPR) assay (see below). Candidate aptamers were subjected to size shortening and optimization by ribose 2′ modifications, giving rise to APT-F2. The APT-F2 is 36-nt in length and contains ribose 2′-O-methyl modifications at 28 positions and 2′-fluoro modifications at five positions.^40^ The SPR analysis indicated that APT-F2 binds stably to FGF2 but not to other FGF family proteins such as FGF1, FGF6, FGF9, and FGF23, as well as heparin-binding proteins such as midkine (MK), pleiotrophin (PTN), epidermal growth factor (EGF), nerve growth factor (NGF) and vascular endothelial growth factor (VEGF) (**Figure 1a**). APT-F2 binds to mouse and rat FGF2 proteins as efficiently as to human FGF2, showing its cross-species reactivity (**Figure 1b**). Importantly, APT-F2 blocked the binding of human FGF2 to its human receptors FGFR1 through FGFR4 when examined with a sensor chip on which the extracellular domains of FGFR fused to IgG-Fc portion were immobilized via the interaction of protein A and Fc (**Figure 1c** and **Supplementary Figure S2a–c**). Furthermore, APT-F2P also blocked the binding of murine FGF2 to its receptors FGFR2 and FGFR3 (see **Supplementary Figure S2d,e**). APT-F2P inhibited the binding FGF2 and the receptor not only human FGF2 but also murine FGF2. Heparin did not interfere with this inhibition.

The dissociation constant (K_D_) of APT-F2 to FGF2 was determined by SPR analysis using a streptavidin-sensor chip on which 3′- or 5′-biotine labeled APT-F2 was immobilized. The running buffer contained a high concentration of salt to reduce nonspecific bindings. Sensorgrams after injection of different concentrations of FGF2 proteins were analyzed and kinetic parameters were estimated (see **Supplementary Figure S3**). APT-F2 has a high affinity to FGF2 with a K_D_ of 17 pmol/l (3′-immobilized) or 34 pmol/l (5′-immobilized).

### Blockade of MAPK/ERK signaling pathway

**Figure 2:**
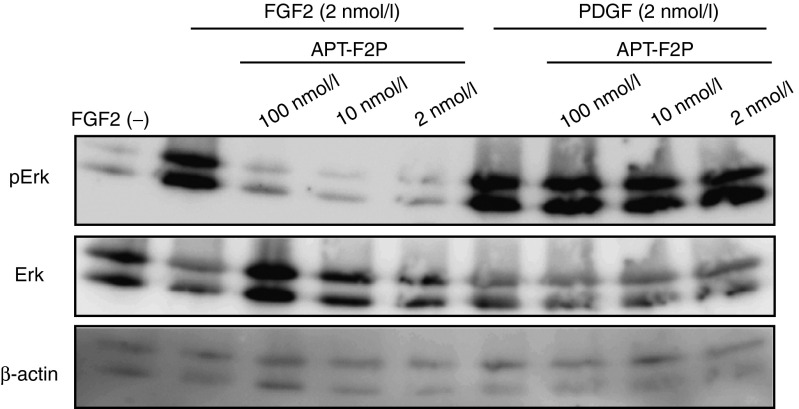
**Inhibition of FGF2-induced signaling pathways in NIH3T3 cells by APT-F2P.** NIH3T3 cells were treated with FGF2 (2 nmol/l) or PDGF (2 nmol/l) with or without APT-F2P (2, 10, and 100 nmol/l) for 0.5 hour, and proteins were analyzed by Western blotting using the indicated antibodies to detect pErk phosphorylation levels and protein loading control (actin and Erk). FGF2, Fibroblast growth factor 2; PDGF, platelet-derived growth factor.

FGF2 can induce the expression of a range of inflammatory cytokines and chemokines^41^ by activating several signaling pathways including MAPK pathway, which leads to the phosphorylation of signaling factors such as ERK.^42^ Since the FGF2 receptor is ubiquitously expressed, almost all cell types have a biological response to FGF2. We examined the effect of APT-F2P, a PEGylated form APT-F2, on FGF2-dependent phosphorylation of ERK1/2. FGF2 (2 nmol/l) treatment of NIH3T3 cells increased phosphorylation of the downstream effector molecule ERK1/2 (**Figure 2**), without significantly affecting total ERK1/2 level. This induced phosphorylation was completely abolished by APT-F2P even at 2 nmol/l concentration, equivalent to the input FGF2 concentration, demonstrating a strong inhibitory potency of APT-F2P (**Figure 2**). On the other hand, APT-F2P did not inhibit ERK1/2 phosphorylation induced by platelet-derived growth factor (PDGF)-BB (**Figure 2**), reinforcing the specificity of APT-F2P.

### Effect of APT-F2P on cellular proliferation, cell viability, and differentiation

**Figure 3:**
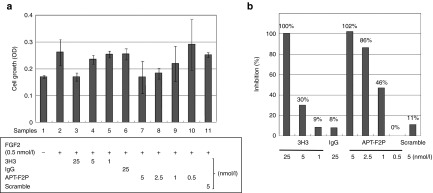
**Attenuation of FGF2-induced proliferation of HUVEC cells by APT-F2P.** (**a**) HUVEC cells growth was examined in the presence of FGF2 (0.5 nmol/l) and the neutralizing antibody 3H3 or APT-F2P at the indicated doses for 72 hours. Scramble denotes a negative control RNA in which the original sequence of APT-F2 was scrambled. (**b**) Inhibition profiles of 3H3 and APT-F2P. Experimental conditions and procedures are described in Methods. Values are the mean and SD of three or four independent experiments. FGF2, Fibroblast growth factor 2; HUVEC, human umbilical vein endothelial cells.

**Figure 4:**
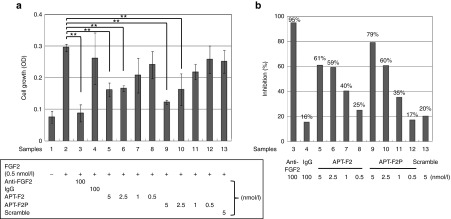
**Attenuation of FGF2-induced proliferation of normal HFLS cells by APT-F2P.** (**a**) HFLS cells growth was examined in the presence of FGF2 (0.5 nmol/l) and the neutralizing anti-FGF2 antibody or APT-F2 or APT-F2P at the indicated doses for 72 hours. (**b**) Inhibition profiles of APT-F2 and APT-F2P. Values are the mean and SD of three or four independent experiments. **P < 0.01 versus HFLS cells growth in the presence of FGF2 (Dunnett's test). FGF2, Fibroblast growth factor 2; HFLS, human fibroblast-like synovial.

FGF2 is capable of stimulating cells proliferation and inducing apoptosis. In this study, we examined the effect of APT-F2 and/or its PEGylated form APT-F2P in different concentrations on FGF2-stimulated cell growth in different cell types. First, human umbilical vein endothelial cells (HUVEC) were examined using APT-F2P and a neutralizing mAb 3H3 as reference (**Figure 3a**). APT-F2P and 3H3 blocked the FGF2-stimulated HUVEC growth in a concentration-dependent manner (**Figure 3b**) with estimated half-maximal inhibitory concentration (IC_50_) values of 1 and 6.5 nmol/l, respectively. The FGF2-induced proliferation of G292 (osteosarcoma cell) was inhibited by APT-F2P more sensitively with an estimated IC_50_ value below 1 nmol/l (see **Supplementary Figure S4**). Second, human fibroblast-like synoviocyte (HFLS) cells derived from normal donors and RA patients were tested. Normal HFLS cells growth stimulated by FGF2 was blocked by both APT-F2 and APT-F2P with IC_50_ values of a few nmol/l range (**Figure 4**). Similarly, the FGF2-simulated growth of HFLS cells derived two RA patients was blocked by APT-F2 and APT-F2P with IC_50_ values of a few nmol/l range (see **Supplementary Figure S5**).

**Figure 5:**
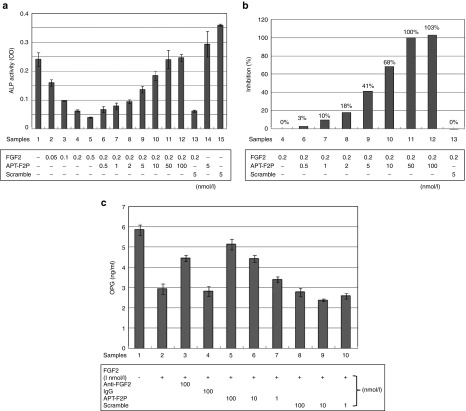
**Effects of APT-F2P on premature osteoblasts differentiation and OPG production in HFLS cells.** (**a**) Attenuation of FGF2-induced differentiation arrest of premature osteoblasts by APT-F2P. MC3T3-E1 cells were grown in minimum essential medium alpha (α-MEM) supplemented 10% FBS, and transferred to the differentiation medium supplemented with the indicated compounds, followed by the ALP activity measurement. Experimental conditions and procedures are described in Methods. (**b**) Inhibition profiles of APT-F2P in FGF2-induced differentiation arrest. (**c**) FGF2 blocks OPG production and APT-F2P liberates the production in HFLS cells. HFLS cells were grown in the presence of FGF2 (1 nmol/l) and the neutralizing anti-FGF2 antibody or APT-F2P at the indicated doses for 72 hours. The secreted OPG level was monitored with ELISA kits (Pierce) using anti-OPG antibody according to the manufacturer's instructions. ALP, alkaline phosphatase; ELISA, enzyme-linked immunosorbent assay; FBS, fetal bovine serum; FGF2, Fibroblast growth factor 2; HFLS, human fibroblast-like synovial; MEM-α, minimum essential medium alpha; OPG, osteoprotegerin.

The effects of FGF2 on osteoblasts are differentiation stage specific. It stimulates premature osteoblast proliferation and inhibits osteoblast differentiation.^8,9,10^ We confirmed that FGF2 stimulated, slightly but significantly, cell growth of premature osteoblasts (MC3T3-E1) and APT-F2P blocked cell growth (see **Supplementary Figure S6**). More profound effects of FGF2 and APT-F2P were observed on osteoblast differentiation. FGF2 sharply inhibited expression of alkaline phosphatase (ALP), a biomarker of osteoblast differentiation, and APT-F2P restored ALP expression by neutralizing the FGF2 activity with an estimated IC_50_ value of 6 nmol/l (**Figure 5a**,**b**).

### APT-F2P liberates OPG synthesis blocked by FGF2 in HFLS cells

It is known that FGF2 inhibits the production of OPG by HFLS cells.^22^ We confirmed in our system that the production of OPG by HFLS cells was blocked by FGF2, and this blockade was liberated by a neutralizing anti-FGF2 mAb and APT-F2P (**Figure 5c**). The IC_50_ of APT-F2P was a few nmol/l range.

### Pharmacokinetic property

APT-F2 of 36-nt in length is heavily modified at its ribose 2′ positions with O-methyl and fluoro modifications to resist ribonucleases. We measured the stability of APT-F2 and APT-F2P in three different human sera of distinct ribonuclease activities. As control, we used the 28-nt non-PEGylated oligonucleotide of Macugen, an anti-VEGF therapeutic RNA aptamer approved for the treatment of age-related macular degeneration,^43^ containing multiple ribose 2′-O-methyl and 2′–fluoro modifications. In the most reactive sera, the half-life (t_1/2_) of APT-F2, APT-F2P and Macugen was estimated 1.4 hours, 2.2 hours, and 17 minutes, respectively (see **Supplementary Figure S7a**). On the other hand, in normal less reactive human sera, APT-F2 and Macugen were stable for 24 hours (see **Supplementary Figure S7b**). Likewise, APT-F2 and Macugen were stable for 24 hours in mouse sera (see **Supplementary Figure S7c**). When APT-F2P was administered intravenously into mice and rats, the t_1/2_ values in blood circulations were 29 and 14 hours, respectively. These findings prompted us to test the efficacy of APT-F2P in the *in vivo* animal models.

### Reduced bone disorders in collagen-induced arthritis mice by APT-F2P

**Figure 6:**
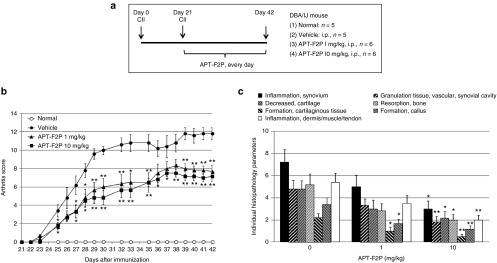
**Attenuation of CIA development in mice by APT-F2P**. (**a**) Procedures of mouse CIA experiments. Experimental details are described in Methods. (**b**) CIA clinical scores for vehicle and APT-F2P administered mice. Values are the mean and SEM of 5–6 mice per group. *P < 0.05 and **P < 0.01 versus vehicle (Mann-Whitney's *U*-test or Dunnett's test). (**c**) Histopathological analysis of the synovitis and bone and cartilage damage. Synovitis is represented by “inflammation in synovium”, “formation of granulation tissue/vascular/synovial cavity”, and “inflammation in dermis/muscle/tendon”, while bone and cartilage damage is represented by “decreased cartilage”, “bone resorption”, “formation of cartilaginous tissue”, and “formation of callus”. CIA, collagen-induced arthritis; SEM, standard error of mean.

Collagen type II (CII) is the major protein in articular cartilage. It is a candidate autoantigen for RA because antibodies and perhaps T cells against CII occur in patients with RA^44,45,46,47^ and because it is arthritogenic in animals.^48^ CIA has thus become the most intensively studied murine model for human inflammatory arthritides.^49^ DBA/1 mice were immunized with CII at day 0 and day 21, and APT-F2P was administered intraperitoneally every day from day 21 to day 42 in two doses of 1 and 10 mg/kg (**Figure 6a**). APT-F2P resulted in moderate, but significant, improvement in the incidence of arthritis and clinical scores of symptoms compared with injection of vehicle control (**Figure 6b**). Although two doses equally reduced the severity of CIA, the histopathological analysis indicated that synovitis and bone and cartilage damage in CIA were reduced more profoundly than arthritis scores by APT-F2P in a dose-dependent manner (**Figure 6c**). These studies indicate that FGF2 inhibition may have therapeutic potential in RA in humans.

### Suppression of arthritis score in glucose-6-phosphate isomerase -induced arthritis by APT-F2P

Glucose-6-phosphate isomerase (GPI) induced RA animal model is authorized extrapolation of human RA pathophysiology. We also examined the efficacy of APT-F2P using this mouse model. APT-F2P was administered intraperitoneally every other day from day 0 to day 14 at two doses of 1 and 5 mg/kg or from day 8 to day 14 at a dose of 5 mg/kg. Administration of APT-F2P from day 0 to day 14 resulted in significant, dose-dependent improvement in the arthritis score of the symptoms as compared with vehicle-treated controls. Postadministration of APT-F2P from day 8 to day 14 showed tendency to reduce apparent clinical symptoms (see **Supplementary Figure S8**). These results suggest that blockade of FGF2 by APT-F2P has both protective and therapeutic activity against GPI-induced arthritis. It is noteworthy, however, that the CIA and GPI scores are largely weighted toward inflammation rather than bone destruction. Hence, it cannot be concluded whether the observed suppression in arthritis scores was primarily due to the aptamer-driven suppression in inflammation or to curing in bone disorders since FGF2 has an angiogenesis activity.^25^

### Restored bone density in ovariectomized rats by APT-F2P

**Figure 7:**
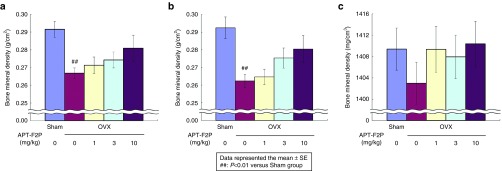
**Blockade of bone density loss in OVX rats by APT-F2P**. (**a,b**) Femur (**a**) and lumbar (**b**) bone mineral density in three months post-OVX measure by DEXA. Values are the mean and SEM of eight mice per group. ##*P* < 0.01 versus Sham group (Student's *t*- test). Experimental conditions and procedures are described in Methods. (**c**) Femur diaphysis bone density in 3 months post-OVX measured by pQCT. DEXA, dual-energy X-ray absorptiometry; OVX, ovariectomized; pQCT, peripheral quantitative computed tomography; SEM, standard error of mean.

Because of the limitation of arthritis models as described above, we examined the effect of APT-F2P in ovariectomized (OVX) rats, a well-established model for osteoporosis, to directly address the effect of APT-F2P on bone since OVX is not associated with inflammation. APT-F2P was administered every other day in three different doses (1, 3, and 10 mg/kg, intravenously) for 90 days into the tail vein of adult female rats immediately following OVX or sham operation. In rats treated immediately post-OVX, OVX produced a 50% decrease in femur and lumbar bone mineral density at day 90 when examined by dual-energy X-ray absorptiometry (DEXA), which was prevented by APT-F2P in a dose-dependent manner (**Figure 7a**,**b**). The restoration of femur bone density was also confirmed by peripheral quantitative computed tomography (pQCT), which provides satisfactory precision and accuracy in skeletal characterization of mouse bones,^50^ showing that even the lowest dose (1 mg/kg) of APT-F2P yielded the normal bone density (**Figure 7c**). In rats 3 months post-OVX, severe bone loss and disruption of trabecular microarchitecture occurred similar to that seen in patients with severe osteoporosis.

Moreover, changes over time of bone metabolic markers, deoxypyridinoline (DPD) and osteocalcin, were assessed using urine and serum samples. Concentration of DPD as a bone resorption marker was measured in collected urine sample during days 24–25, 60–61, and 90–91. Concentration of osteocalcin as a bone formation marker in serum was also measured on days 25, 61, and 91 after oophorectomy. 10 mg/kg administration of APT-F2P significantly inhibited the concentration of DPD in urine on both the days 60–61 and 90–91. Furthermore, the inhibitory effects were shown tendency dose-dependent manner. In terms of osteocalcin in serum, our compound also inhibited the production on day 91 with a statistically significant difference in comparison with vehicle control. These results indicated that APT-F2P suppressed bone metabolic turnover, thereby inhibited the bone loss in OVX rat model (see **Supplementary Figure S9**).

We demonstrated histopathological analysis of bone cell and epiphyseal growth plate. In terms of bone cell condition, APT-F2P showed tendency to inhibit osteoclast cell proliferation upregulated in femur cortical bone of OVX rats (see **Supplementary Figure S10a**). On the other hand, APT-F2P stimulated osteoblast cell proliferation (see **Supplementary Figure S10b**). These findings are interpreted as indicating that APT-F2P acts on osteoclast and osteoblast cells in bone, resulting in suppression of bone mineral density loss in OVX rats. Moreover, the analysis pointed out the severe disruption of the epiphyseal growth plate in OVX rats (see **Supplementary Figure S11a**). Surprisingly, APT-F2P blocked the disruption of the epiphyseal growth plate and restored the bone quality in a dose-dependent manner (see **Supplementary Figure S11b–d**) and 10 mg/kg dose of APT-F2P perfectly reversed the epiphyseal growth plate, equivalent to the Sham control.

### Analgesic activity of APT-F2P in murine femur bone cancer model

**Figure 8:**
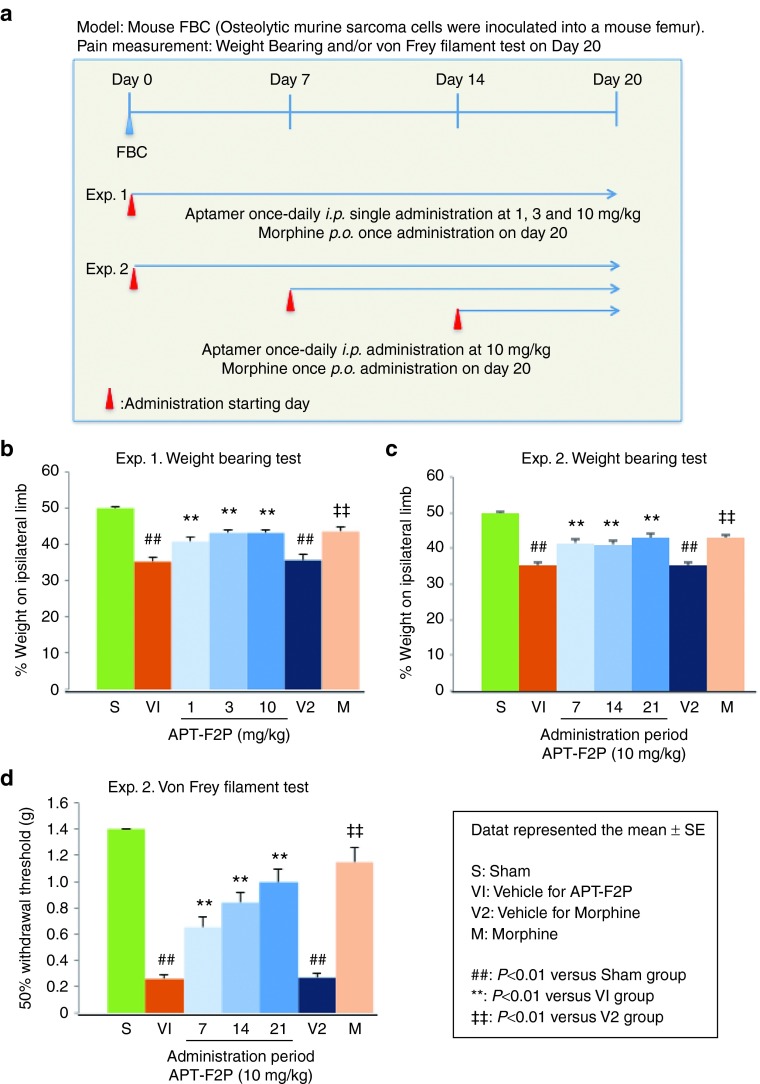
**Analgesic impact on mouse FBC pain**. (**a**) Procedures of mouse FBC pain experiments. Experimental details are described in Methods. (**b,c**) Weight-bearing tests in Experiments 1 and 2, respectively. (**d**) von Frey filament test in Experiment 2. Values are the mean and SEM of 7–13 mice per group. S, V1, V2, and M were represented sham, vehicle for APT-F2P, vehicle for morphine and morphine administration groups, respectively. ##*P* < 0.01 versus S (Aspin Welch's *t*-test), **P < 0.01 versus V1 (Dunnett's test), **††***P* < 0.01 versus V2 (Aspin Welch's *t*-test). FBC, femur bone cancer; SEM, standard error of mean.

Bone diseases are often associated with pain as in bone cancer. In this study, we examined the analgesic activity of APT-F2P in the femur bone cancer (FBC) mouse model, in which ongoing and movement-evoked pain behaviors increase in severity with time and are correlated with the tumor growth and progressive tumor-induced bone destruction.^51,52^ Mouse osteosarcoma tumor cells were injected and confined to the intramedullary space of the mouse femur of left leg, and APT-F2P was administered intraperitoneally every day for 3 weeks (from day 1 to day 20) at three doses of 1, 3, and 10 mg/kg (Experiment 1) or for two (from day 7 to day 20) or one (from day 14 to day 20) week at a dose of 10 mg/kg (Experiment 2) (**Figure 8a**). As a positive control, morphine (10 mg/kg) was orally administered on day 20. Ongoing pain was analyzed on day 20 by the weight-bearing test using the incapacitance device. Three weeks daily administration of APT-F2P significantly (*P* < 0.01) decreased hind paw weight distribution in a dose-dependent fashion, equivalently to morphine (**Figure 8b**). Importantly, late administration of APT-F2P from day 14 to day 20 also significantly (*P* < 0.01) decreased hind paw weight distribution (**Figure 8c**). Allodynia was analyzed by the von Frey filament test, in which the mouse hind paw withdrawal threshold was monitored as the sensation became painful due to injury.^53^ APT-F2P significantly (*P* < 0.01) increased withdrawal threshold of an injured paw in proportion to the administration period, closer to the morphine level (**Figure 8d**). These results demonstrated a strong analgesic action of APT-F2P in cancer pain. The histopathological analysis revealed that APT-F2P did not suppress tumor proliferation and osteolysis of cancer cells inoculated in femur bone (see **Supplementary Figure S12**). Therefore, the analgesic action of APT-F2P in bone cancer pain cannot be accounted for by the suppression of tumor proliferation and osteolysis but by some other mechanism involving a novel FGF2 function in pain.

We further investigated the analgesic potency of APT-F2P in a rat model of postoperative pain. An incision of the plantaris muscle of a hind paw induced spontaneous pain and tactile allodynia lasting a few days. Postoperative testing was carried out using the weight-bearing test for spontaneous pain and the von Frey filament test for tactile allodynia. A single oral dose of pregabalin (30 mg/kg), 4 hours after surgery, completely blocked the development of spontaneous pain and allodynia tested 5 and 8 hours after surgery (see **Supplementary Figure S13**). On the other hand, a single intravenous dose of APT-F2P (3 and 30 mg/kg), 1 hour before surgery, did not prevent the development of spontaneous pain and allodynia (see **Supplementary Figure S13**). These findings are interpreted as indicating that a FGF2 antagonist may not be effective in the treatment of postoperative pain probably because FGF2 is not overproduced immediately following operation.

## Discussion

FGF2 is a multifunctional growth factor involved in tissue remodeling and regeneration, and exerts distinct actions on bone, depending on the developmental stage and disease conditions. It plays a crucial *positive* role in bone remodeling,^1,2^ while the *in vitro* cell-based analysis suggested a severe *negative* role in bone diseases.^54^ In this study, we generated a neutralizing anti-FGF2 aptamer, APT-F2P, and demonstrated that sequestering FGF2 activity by APT-F2P significantly reduced severity of joint disease and bone destruction in CIA, GPI mice, and OVX rats, and further ameliorated bone cancer pain in FBC mice. These results strongly suggest that FGF2 enhances bone disease progression as previously suggested.^54^

The prophylactic effect of APT-F2P on bone density loss in OVX rats are apparently inconsistent with the previous observations.^55,56^ Liang *et al.* reported that vehicle-treated OVX rats exhibited significantly lower cancellous bone volumes and administration of FGF2 resulted in partial restoration of the loss of cancellous bone mass in the osteopenic OVX rats by stimulating bone formation.^56^ They also reported a bone mineralization defect in OVX rats, though the mechanism for impaired bone mineralization was unclear.^56^ Eda *et al.* also reported that FGF2 inhibits bone mineralization and stimulates cell proliferation.^9^ Taking these and other results into consideration, we assume that administration of APT-F2P eliminated the FGF2 blockade and enhanced a bone mineralization, resulting in restoration of bone density. Nevertheless, it cannot be excluded at present that the observed differences might be due to different experimental procedures or conditions.

Although several studies have suggested that FGF2 may be a possible therapeutic target to treat subjects with RA or other bone diseases,^54^ no anti-FGF2 mAb has been developed clinically. There seemed to be two major reasons for this lack of development activity. The first neutralizing mAbs to FGF2 were described in 1989–1991, but the role of FGF2 in bone diseases had long been unknown, and the discrepant reports were made on the role of FGF2 in bone disorders in 1998–2002 as described above. These might have impeded the therapeutic development of FGF2 antagonists to treat bone diseases. Moreover, although a number of anti-FGF2 mAbs have been developed and shown to neutralize various activities of FGF2 *in vitro* and in some cases *in vivo*, most of them seemed to be inadequate for therapeutic development. Only recently, anti-FGF2 mAb called GAL-F2 has been developed for anticancer therapeutics, which is likely to recognize, unusually, two separate epitopes on the surface of FGF2.^35^ It is generally uneasy to raise an antibody to recognize two separate epitopes on the target protein. On the other hand, the isolation of aptamers that recognize separate epitopes on the surface is relatively easy since aptamers generally capture the shape of the target protein.

There are no universally accepted agents that will substantially cure bone disorders in osteoporotic patients. The present findings suggest that APT-F2P merits consideration for development as a potential treatment for patients with severe osteopenia who are unresponsive to conventional osteoporosis therapies. In this study, to our surprise, the severe disruption of the epiphyseal growth plate occurred in OVX rats, and APT-F2P sharply blocked the disruption of the epiphyseal growth plate in a dose-dependent manner. 10 mg/kg dose of APT-F2P completely reversed the epiphyseal growth plate, equivalent to the Sham control. It has been shown previously that overexpression of FGF2 in transgenic mice resulted in achondroplasia and shortening of the long bones.^57^ Achondroplasia is a rare genetic disease characterized by abnormal bone development, resulting in short stature. It is caused by a single point mutation in the gene coding for FGFR3, which leads to prolonged activation upon ligand binding.^58^ These independent observations can be interpreted as indicating that FGF2 might be overexpressed in OVX rats, leading to impaired epiphyseal growth plate, and the blockade of FGF2 action by APT-F2P ameliorates the epiphyseal growth plate and might cure achondroplasia. Therefore, APT-F2P should have a significant impact in therapeutics to prevent skeletal dysplasias, including achondroplasia, related to excessive FGFR activation. In this regard, it is worth mentioning that Garcia *et al.* have developed a recombinant protein therapeutic approach using a soluble form of human FGFR3 to prevent achondroplasia, which acts as a decoy receptor and prevents FGF from binding to mutant FGFR3.^59^

Bone cancer pain is one of the most common cancer-related pains, and appears to be driven simultaneously by inflammatory, neuropathic, and tumorigenic mechanisms, resulting in deep pain with a burning and stabbing sensation often described by bone cancer patients.^60^ NGF is known to transmit peripheral pain signal to brain and has been shown to modulate inflammatory and neuropathic pain states. The blockade of NGF activity by a neutralizing antibody to NGF produced a profound reduction in both ongoing and movement-evoked bone cancer pain-related behaviors.^61^ However, there is no literature that relates FGF2 to bone cancer pain. In this study, APT-F2P exerted a strong analgesic impact, equivalent to morphine, in a mouse model of bone cancer pain. To our knowledge, this is the first report demonstrating direct *in vivo* evidence of pain-modulatory effects of FGF2 in bone cancer pain. We assume that not only NGF but also FGF2 might be overexpressed by bone cancer. Since FGF2 is known to stimulate angiogenesis and endogenous VEGF expression,^62^ excess FGF2 and VEGF can synergize to induce angiogenesis and increase severity of bone cancer progression. One might speculate that sequestering FGF2 activity by APT-F2P might reduce endogenous NGF and produce analgesia in view of the published reports that FGF2 stimulates NGF expression.^63,64^ However, this is unlikely since APT-F2P was ineffective in a rat model of postoperative pain as shown in this study. This is in sharp contrast to the action of anti-NGF aptamer, which was effective to attenuate postoperative pain in a rat model (unpublished). Hence, the pathophysiological role of FGF2 in bone cancer pain is largely independent from that of NGF. Furthermore, the analgesic action of APT-F2P in bone cancer pain cannot be accounted for by the suppression of tumor proliferation and osteolysis. Precise pain-modulatory mechanism of FGF2 remains to be investigated. Taking these results into consideration, we assume that APT-F2P might be effective in ameliorating chronic pain associated with FGF2 overproduction, such as in bone cancer, but not acute pain unassociated with FGF2 overproduction, such as in immediate postoperation.

Since the pathophysiology of RA has been thought to involve synovial proliferation and angiogenesis as well as bone destruction and absorption, blockade of VEGF, dominantly resulting in suppression of angiogenesis and fluid collection, might not be sufficient to prevent RA disease from the viewpoint of clinical treatment. Our current results suggest that FGF2 seems to be a better molecular target of RA, because it might indirectly control VEGF expression via regulating FGF2. More importantly, APT-F2P might provide us with a curative therapy for bone diseases by liberating OPG production, blocking RANK ligand expression, stimulating osteoblast differentiation, and preventing osteoclastogenesis in murine bone marrow cultures. Collectively, dual potency of APT-F2P, not only by blocking pain but also by curing bone disorders, should be an attractive therapeutic advantage. This is in sharp contrast to currently available or developing therapeutic antibodies to NGF^65^ or to RANK ligand,^66^ which is effective only in pain or bone disorders. Moreover, FGF2 is believed to play a role in cancer, both by stimulating angiogenesis and tumor cells growth directly. Therefore, APT-F2P should have another merit of blocking tumor progression in bone cancer. These perspectives remain to be addressed in clinical studies. To our knowledge, this study provides the first *in vivo* evidence that FGF2 is involved in bone disease progression.

## Materials and Methods

***Materials.*** APT-F2P used for animal experiments was 5′ and 3′ conjugated with 40-kDa PEG (SUNBRIGHT GL2-400TS, NOF Corporation, Tokyo, Japan) and an inverted dT (idT), respectively, were prepared by chemical synthesis (Gene Design, Ibaraki, Japan). Growth factors used are: recombinant human FGF1 (PEPROTECH, Rocky Hill, KY), human FGF2 (PEPROTECH), human FGF6 (R&D Systems, Minneapolis, MN), human FGF9 (R&D Systems), human FGF23 (R&D Systems), human PDGF-BB (PEPROTECH), human EGF (PEPROTECH), human VEGF (PEPROTECH), human NGF (R&D Systems), human MK (PEPTIDE INSTITUTE, Ibaraki, Japan), human PTN (PEPTIDE INSTITUTE), murine FGF2 (PEPROTECH), and rat FGF2 (PEPROTECH). FGF receptor/Fc fusions used are: human recombinant FGFR1α(IIIc)-Fc (R&D Systems), human FGFR2β(IIIc)-Fc (R&D Systems), human FGFR3(IIIc)-Fc (R&D Systems), human FGFR4-Fc (R&D Systems), mouse recombinant FGF R2β(IIIc)-Fc (R&D Systems) and mouse recombinant FGF R3(IIIc)-Fc (R&D Systems). Heparin was purchased from Calbiochem, San Diego, CA. Antibodies used are: neutralizing anti-FGF2 mAb “3H3” (Calbiochem), neutralizing anti-FGF2 mAb (R&D Systems), anti-phospho-(p)Erk 1/2 (Thr202/Tyr204) mAb (Cell Signaling Technology, Danvers, MA), anti-Erk (p44/42 MAP kinase) mAb (Cell Signaling Technology), anti-β-actin mAb (Sigma-Aldrich, St Louis, MO), normal goat IgG (R&D Systems), and antirabbit IgG-HRP antibody (Jackson Immuno Research Laboratories, West Grove, PA). Materials used for OPG assay are: antihuman OPG/TNFRSF11B antibody (R&D Systems, CUZ04, as capture antibody), antihuman OPG/TNFRSF11B-biotinylated antibody (R&D Systems, BAF805, as detection antibody), recombinant human OPG/TNFRSF11B (R&D Systems, as standard), and streptavidin-HRP (R&D Systems). Cell lines used are: NIH3T3 (DS Pharma Biomedical, Suita, Japan), HUVEC (CAMBREX, East Rutherford, NJ), normal adult HFLS (Cell Applications, San Diego, CA), RA-patient adult HFLS (Cell Applications), G292 (DS Pharma Biomedical), and MC3T3-E1 (RIKEN Cell Bank, Tsukuba, Japan).

***Animals.*** Male DBA/1 mice were obtained from Charles River Japan (Yokohama, Japan). Male C3H/HeJYokSlc mice were obtained from Japan SLC, Hamamatsu, Japan. Female Sprague-Dawley (SD) rats were obtained from Charles River Japan. Mice and rats were maintained under special pathogen-free conditions. Animal experiments were performed in accordance with the Guidelines for Animal Experiments of the Institute of Medical Science, University of Tokyo (Japan), Hamamatsu Pharma Research, Hamamatsu, Japan, and Shin Nippon Biomedical Laboratories, Tokyo, Japan, Experimental protocols of mouse CIA and GPI models were approved by the animal experiment committee of the Institute of Medical Science, University of Tokyo, and the protocol of OVX rat model experiment was approved by the animal experiment and licensing committee of Shin Nippon Biomedical Laboratories. Experimental protocols of cancer pain mouse model and incisional pain rat model were approved by the animal experiment and licensing committee of Hamamatsu Pharma Research.

***Selection of aptamers.*** SELEX was carried out essentially as described as a primer-less SELEX^39^ with some modifications. The sequence of single-stranded DNA (ssDNA) template was 5′5′-TCGAG-30N-TCCC*TATAGTGAGTCGTATTA*-3′, where 30N represents 30-nt random sequence and the complementary sequence of T7 promoter is underlined. The ssDNA template was hybridized with the forward (T7 promoter) primer (5′-AAGCCTGTGGAGCTGC*TAATACGACTCACTATA*GGGA-3′, where the T7 promoter sequence is underlined), and the random N30 RNA pools (5′-GGGA-30N-CTCGA-3′transcripts) were synthesized by DuraScribe T7 RNA transcription kit (Epicentre, Madison, WI) using 2′-fluoro-CTP and 2′-fluoro-UTP under 10 molar excess condition of GMP relative to GTP (to generate 5′-mono-phosphate terminus accessible to ligation). The resulting random sequence pools of 5′-GGGA-30N-CTCGA-3′transcripts were used for affinity selection to human FGF2 essentially as described previously^67^ except that the target FGF2 was immobilized to NHS-activated Sepharose 4B resin. For the subsequent round of selection and amplification, the T7 promoter sequence (5′-TAATACGACTCACTATA-3′) was ligated to the 5′ terminus of the selected RNA sequences in the presence of the forward bridge sequence (5′-TCCCTATAGTGAGTCGTATTA-NH2-3′), and the 3′ terminus of the selected RNA sequences was ligated to the reverse adaptor sequence (5′-p-GAGAACTAAGCTGAACAAGA-NH2-3′) in the presence of the reverse primer sequence (5′-TCTTGTTCAGCTTAGTTCTCTCGAG-3′). The resulting sequence extension was amplified with forward (5′-AAGCCTGTGGAGCTGC*TAATACGACTCACTATA*GGGA-3′, where the T7 promoter sequence is underlined) and reverse (5′-TCTTGTTCAGCTTAGTTCTCTCGAG-3′) primers using Ex Taq polymerase (Takara, Kusatsu, Japan). The amplified double-stranded DNAs (dsDNAs) were digested by *Xho*I restriction enzyme to regenerate the original 3′-terminal structure of the DNA template, and selected RNA sequences were synthesized and subjected to SELEX as described above. Oligonucleotides used in this study were prepared by chemical synthesis (Gene Design).

***Manipulations of aptamers.*** Of 400 RNAs selected by SELEX, the lead aptamer that gave rise to the final candidate APT-F2 was 38-nt in length and contained 2′-fluoro pyrimidines. The lead aptamer was shortened to 36-nt without losing the activity, and ribose 2′ positions were systematically examined for O-methyl modifications to confer the resistance to ribonucleases and to increase the activity to block FGF2 function. The APT-F2 thus constructed is 36-nt in length and contains ribose 2′-O-methyl modifications at 28 positions and 2′-fluoro modifications at five positions, leaving three positions unmodified.^40^

***SPR assay.*** The SPR assays were performed essentially as described previously using the Biacore 2000 instrument (GE Health Care Life Sciences, Uppsala, Sweden).^67^ The RNA monoclones selected by SELEX were prepared by using the DuraScribe T7 Transcription Kit. The affinity of these RNAs to FGF2 was analyzed with a CM4 sensor chip, on which ~2,000–4,000 RU of FGF2 were immobilized by amino-coupling. The running buffer was a mixture of 295 mmol/l NaCl, 5.4 mmol/l KCl, 0.8 mmol/l MgCl_2_, 1.8 mmol/l CaCl_2_, 20 mmol/l Tris (ph7.6), and 0.05% Tween20. A high concentration of NaCl was used due to reduce nonspecific bindings. A 2 mol/l NaCl solution was used for regeneration of the sensor chip. In experiments of the binding specificity and the kinetics, the biotinylated APT-F2 was immobilized onto a SA sensor chip and proteins were injected. The K_D_ values were estimated by using BIAevaluation 3.0 software. To assess the human and mouse FGF2-FGFR interaction, FGFR/Fc fusion proteins were immobilized onto a CM5 sensor chip via prebound protein A (Pierce) (1,000 RUs), and FGF2 was injected with or without the APT-F2 aptamer.

***Cell viability assay.*** HUVECs were cultured in EGM-plus growth medium (Lonza, Walkersville, MD) at 37°C in humidified air with 5% CO_2_, and were seeded at 5 × 10^3^ cells/well into 96-well microtiter culture plates. After incubation for 24 hours, cells were washed twice with 1× phosphate-buffered saline (PBS) (pH7.4), and cultured in the same medium supplemented with FGF2, APT-F2P or a neutralizing mAb 3H3 (Calbiochem) at the indicated concentrations for 3 days. G292 cells were cultured in Roswell Park Memorial Institute medium (RPMI) -1640 (Gibco) containing 0.5% fetal bovine serum (FBS), 100 U/ml penicillin and 100 µg/ml streptomycin at 37°C in humidified air with 5% CO_2_, and were seeded at 2 × 10^3^ cells/well into 96-well microtiter culture plates. After incubation for 30 minutes, cells were washed twice with 1 × PBS (pH7.4), and cultured in the same medium supplemented with FGF2, APT-F2P or a neutralizing mAb (R&D Systems, note that 3H3 mAb is used only for the HUVEC cell growth test) at the indicated concentrations for 3 days. HFLS cells were cultured in synoviocyte growth medium (Cell Application) at 37°C in humidified air with 5% CO_2_, and were seeded at 5 × 10^3^ cells/well into 96-well microtiter culture plates. After incubation for 30 minutes, cells were washed twice with 1×PBS (pH7.4), and cultured in the same medium supplemented with FGF2, APT-F2P or a neutralizing antibody at the indicated concentrations for 3 days. MC3T3-E1 cells were cultured in minimum essential medium alpha (α-MEM) (Gibco, Grand Island, NY) supplemented 10% FBS, 100 U/ml penicillin and 100 µg/ml streptomycin at37°C in humidified air with 5% CO_2_, and seeded at 2 × 10^4^ cells/well into 96-well microtiter culture plates. After incubation for 30 minutes, cells were washed twice with 1× PBS (pH7.4) and cultured 2% FBS in α-MEM supplemented with FGF2 and APT-F2P or neutralizing mAb (R&D Systems) for 3 days. Cells proliferation was determined by monitoring cell viability with Cell Counting Kit-8 (Sigma) according to the manufacturers' instructions.

The viable cell count was read by optical density (OD) at 450 nm. Inhibitory effect and IC_50_ were calculated as following formula from the obtained OD_450_ values:

% Inhibition = 100 × ((OD_450_ value in the presence of FGF2) – (OD_450_ value in the presence of FGF2 and APT-F2P)) / ((OD_450_ value in the presence of FGF2) – (OD_450_ value in the absence of FGF2))

IC_50_ of APT-F2P was determined by drawing inhibition curve.

***FGF2 or PDGF-BB induced signaling assay.*** NIH-3T3 were cultured in Dulbecco's modified essential medium (DMEM) (Gibco) supplemented with 10% FBS, 100 U/ml penicillin and 100 µg/ml streptomycin at 37°C in humidified air with 5% CO_2_, and were seeded at 2.5 × 10^4^ cells/well into 96-well microtiter culture plates. After incubation for 6 hours, cells were subsequently cultured in FBS-free DMEM medium for overnight. Then, cells were treated with FGF2 and PDGF-BB and APT-F2P at the indicated concentrations for 30 minutes, and subjected to cell lysis and Western blotting using phosphor-specific antibody, P-ERK T202/Y204. The β-actin was used as a control.

***OPG production assay.*** HFLS cells were used in the assay. HFLS cells (8 × 10^3^ cells/well) were seeded in 96-well microtiter plate, incubated for 30 minutes, and treated with FGF2 (1 nmol/l) and APT-F2P (1, 10, and 100 nmol/l). After 3 days, OPG production quantity in culture supernatant was evaluated using the enzyme-linked immunosorbent assay (ELISA) kit (R&D) according to the manufacturer's instructions.

***Cell differentiation assay.*** MC3T3-E1 cells were cultured in minimum essential medium alpha (α-MEM) (Gibco) supplemented 10% FBS, 100 U/ml penicillin and 100 µg/ml streptomycin at 37°C in humidified air with 5% CO_2_, and seeded at 2 × 10^4^ cells/well into 96-well microtiter culture plates. After incubation for 24 hours, cells were transferred to differentiation medium containing 0.2% (v/v) hydrocortisone, 2% (v/v) 2-glycerol phosphate, and 1% (v/v) ascorbic acid in α-MEM, supplemented with FGF2 and APT-F2P at the indicated concentrations. Medium was exchanged at 3-day intervals, and after 8 days culture, ALP activity was measured using the ALP Assay Kit (TaKaRa) with p-nitro-phenyl phosphate as substrate according to the manufacturer's instructions. The ALP activity was measured by monitoring OD at 405 nm. Inhibitory effect and IC_50_ were calculated as following formula from the obtained OD_405_ values:

% Inhibition = 100 × ((OD_405_ value in the presence of FGF2 and APT-F2P) – (OD_405_ value in the presence of FGF2)) / ((OD_405_ value in the absence of FGF2) – (OD_405_ value in the presence of FGF2))

IC_50_ of APT-F2P was determined by drawing inhibition curve.

***CIA mouse model.*** DBA/1 mice were immunized intradermally at day 0 and day 21 with 0.1 mg of Collagen type II (CII) in Complete Freund's adjuvant (Chondrex, Redmond, WA) emulsified at a 1:1 ratio (volume/volume in 0.1 ml) into the base of the tail. For the control, PBS and Complete Freund's adjuvant were emulsified and injected (0.1 ml). APT-F2P was administered intraperitoneally every day from day 21 to day 42 in two doses of 1 and 10 mg/kg. The clinical arthritis was assessed beginning from day 21 of primary immunization. The severity of arthritis in each of the four paws was graded in a scale of 0–4 as follows: 0, no evidence of erythema and swelling; 1, erythema and mild swelling confined to the tarsals or ankle joint; 2, erythema and mild swelling extending from the ankle to the tarsals; 3, erythema and moderate swelling extending from ankle to metatarsal joints; and 4, erythema and severe swelling encompass the ankle, foot and digits, or ankylosis of the limb. Four limbs were graded and totally scores were expressed in each animal. For histopathological analysis, four joints of the CIA mice were fixed in phosphate-buffered 10% formaldehyde, decalcified, and each limb was processed in paraffin blocks and sectioned at 5 μm in thickness at the part, which is from the wrist to the index finger. Sections were stained with hematoxylin and eosin and examined by light microscopy. Scoring (0–3) of “inflammation in synovium”, “formation of granulation tissue/vascular/synovial cavity”, “inflammation in dermis/muscle/tendon”, “decreased cartilage”, “bone resorption”, “formation of cartilaginous tissue”, and “formation of callus” was performed blind evaluation by a single pathologist.

***Measurement of bone turnover markers in urine and serum.*** Urine samples were collected during days 24–25, 60–61, and 90–91 after oophorectomy using metabolic cage. Serum samples also were collected on days 24, 60, and 90. Until analysis, these samples were stored at −80°C. Concentrations of DPD in urine and osteocalcin in serum were determined by using ELISA Kits, Osteolinks “DPD” (Quidel Corporation, San Diego, CA), and Rat Osteocalcin ELISA Kit (Immutopics, San Clemente, CA), respectively.

***GPI-induced mouse model.*** DBA/1 mice were immunized intradermally at day 0 with 0.2 mg of recombinant human GPI (hGPI) in Complete Freund's adjuvant (Difco, Lawrence, KS) emulsified at a 1:1 ratio (volume/volume) into the base of the tail. APT-F2P was administered intraperitoneally every other day from day 0 to day 14 at two doses of 1 and 5 mg/kg or from day 8 to day 14 at a dose of 5 mg/kg. The severity of arthritis in each of the four paws was graded in a scale of 0–4 identical to the CIA mouse model. Four limbs were graded and totally scores were expressed in each animal.

***OVX rat model.*** Adult female SD rats (300–350 g), aged 19 months, were sham-operated or OVX. On the day of surgery, all rats were performed under deep (2.0~4.0%) isoflurane anesthesia conditions. Eight rats were subjected to sham surgery, during which the ovaries were exteriorized but replaced intact. Bilateral ovariectomies were performed in the remaining 32 rats on day 0. APT-F2P was administered intravenously every other day from day 3 to day 90 in three doses of 1, 3, and 10 mg/kg. All rats were sacrificed on day 91 by exsanguination from the abdominal aorta under anesthesia from an intraperitoneal injection of sodium pentobarbital (Tokyo Chemical Industry, Tokyo, Japan) solution (6.48 and 5 ml/kg). At necropsy, failure to detect ovarian tissue and observation of marked atrophy of the uterine horns confirmed the success of ovariectomy. After as much of muscle and soft tissue as possible were removed from femur and lumbar were fixed in 10% neutral buffered formalin. Each bone was wrapped in saline-soaked gauze and stored in a histo-pack until measurement in a freezer. The bone mineral density of femur and lumbar from each animal was examined by DEXA and pQCT. The histopathological analysis of the bone was carried out essentially as previously described.^55^ Briefly, the femur was fixed in 10% neutral buffered formalin, decalcified, embedded in paraffin, and sectioned at 5 μm in thickness. Sections were stained with hematoxylin and eosin and examined under light microscopy. The pathological findings of “increasing osteoclast” and “increasing osteoblast” in the femur were showed score (0–4). Some additional sections were stained with 1% toluidine blue for microscopic observation. The morphological analysis was performed in cancellous bone tissue of the femur metaphysis, beginning at a distance of 1 mm from the growth plate-metaphyseal junction to exclude the primary spongiosa. Scoring (0–4) of decreased toluidine blue stained area in the growth plate and metaphysis was performed blind evaluation by a single pathologist.

***Culture and injection of tumor cells.*** Osteolytic murine sarcoma cultured cells (NCTC 2472, ATCC, Rockville, MD) were inoculated into the femur of left leg of male C3H/HeJYokSlc mice as previously described.^61,68^ In brief, following induction of general anesthesia with sodium pentobarbital (50 mg/kg, intraperitoneally), an arthrotomy was performed exposing the condyles of the distal femur. D-MEM/F12 medium containing 10^5^ osteolytic murine sarcoma cells (10 µl) was inoculated into the intramedullary space of the mouse femur. APT-F2P was administered intraperitoneally every day for 3 weeks (Experiment 1) or for 1 or 2 weeks (Experiment 2) postinoculation. A day 20 endpoint was used, as this is the time point when the tumor is still confined to the bone and exhibits maximal presentation of cancer-related pain behaviors. For histopathological analysis, murine sarcoma cells inoculated femur were fixed in phosphate-buffered 10% formaldehyde, decalcified, and each femur was processed in paraffin blocks and sectioned at 5 μm in thickness. Sections were stained with hematoxylin and eosin and examined by light microscopy. Scoring (0–4) of “proliferation of tumor cell” and “osteolysis” was performed blind evaluation by a single pathologist.

***Incisional pain rat model.*** The incisional pain model was prepared according to Brennan's method.^69^ Under isoflurane anesthesia, a 1-cm longitudinal incision was made through skin, fascia, and muscle of the plantar aspect of the left foot. After hemostasis with gentle pressure, the skin was sutured closed with a 5-0 nylon thread. Normal rats were anesthetized only. APT-F2P was administered intravenously 1 hour before incision at doses of 3 and 30 mg/kg. Pregabalin as a positive compound was administrated orally 4 hours after incision at dose of 30 mg/kg. Measurement of pain threshold using weight bearing and von Frey filament tests were demonstrated 5 and 8 hours postincision.

***Weight-bearing test.*** The weight-bearing test represents an unsurpassed method for assessing spontaneous pain and postural deficits in laboratory animals. The test animals were placed in the incapacitance device, which quantifies the spontaneous postural changes reflecting spontaneous pain by independently measuring the weight that the animal applies each hind paw on two separate sensors. Normal mice distribute weight equally on both paws, and change of this equilibrium can reflect the level of discomfort due to an injured paw. Briefly, prior to evaluation, mice were acclimated once to the incapacitance device. Weight bearing on the hind limbs was determined using an incapacitance tester. Mice were placed in the test chamber and held in the appropriate position in the incapacitance tester and measured. After each animal underwent three recordings, the mean percent (%) weight bearing on the ipsilateral (injected) limb was calculated. If an animal moved during the measurement, the value was excluded and measured again. The ratio of weight bearing on the ipsilateral limb was calculated by the following formulas.

The ratio of weight bearing on the ipsilateral limb (%) = 100 × (weight on the ipsilateral limb)/((weight on the ipsilateral limb) + (weight on the contralateral limb))

***von Frey filament test.*** von Frey filaments rely on the principle that an elastic column, in compression, will buckle elastically at a specific force, dependent on the length, diameter, and modulus of the material. The filaments are used to provide a range of forces to the skin of a test subject, in order to find the force at which the subject reacts because the sensation is painful.^53^ Briefly, prior to evaluation, mice were acclimated once to the testing environment. On the measurement day 2.5–3 hours after final administration, the mouse was placed in a plastic chamber with a wire mesh floor for ~30 minutes. Measurement was started after confirming that the mouse was calm. The 50% withdrawal threshold to mechanical stimulation was determined utilizing a protocol described by Chaplan *et al.*^53^ by using von Frey filaments. von Frey filaments (bending force: 0.02, 0.04, 0.07, 0.16, 0.4, 0.6, 1.0, and 1.4 g; starting at 0.16 g) were applied perpendicularly to the plantar midsurface of the left (injected) hind paw. The filament was bent for ~4 seconds and the occurrence of a withdrawal response was noted. When no response was observed a higher filament was used. If a response was observed, a lower filament was used. After paw withdrawal responses to von Frey stimulation were measured, the 50% threshold was calculated by the Dixon's up-down method.^70^

***Statistical analysis.*** All data regarding *in vivo* experiments were showed mean and standard error of mean that were calculated by Microsoft Excel. The statistical analysis was demonstrated by following software in each experiment. The clinical scores and histopathological scores were analyzed using Ekuseru-Toukei 2006 for Windows (Social Survey Research Information, Tokyo, Japan). The bone mineral density of femur and lumbar, 50% withdrawal threshold and % weigh of the ipsilateral limb were analyzed using SAS System for Windows, Release 9.2 or 9.3 (SAS Institute, Cary, NC). Differences between vehicle and administrated groups were considered statistically significant at *P* < 0.05.

**SUPPLEMENTARY MATERIAL** **Figure S1.** A schematic illustration of the action of FGF2 in bone disease progression. **Figure S2.** SPR sensorgrams showing the ability of APT-F2 to block the interaction of human FGF2 with human FGFR2, FGFR3, and FGFR4. **Figure S3.** Binding profile of APT-F2 measured by SPR. **Figure S4.** Attenuation of FGF2-induced proliferation of G292 cells by APT-F2P. **Figure S5.** APT-F2P mediated attenuation of FGF2-induced proliferation of HFLS cells derived from two RA patients. **Figure S6.** Attenuation of FGF2-induced proliferation of premature osteoblasts by APT-F2P. **Figure S7.** Stability of APT-F2 and APT-F2P in different kinds of sera. **Figure S8.** Development of GPI-induced arthritis is suppressed by APT-F2P in mice. **Figure S9.** Inhibitory effects of APT-F2P on secretion of urinary DPD and serum osteocalcin. **Figure S10.** Cell numbers of osteoclasts and osteoblasts in femoral bones of OVX rats untreated and treated with APT-F2. **Figure S11.** Bone histology in OVX rats. **Figure S12.** Histopathological assessment of tumor proliferation and osteolysis in FBC pain model. **Figure S13.** Effects of APT-F2P on a rat model of postoperative pain.
