# Supplementary material for: Dual Therapeutic Action of a Neutralizing Anti-FGF2 Aptamer in Bone Disease and Bone Cancer Pain
Source: Mol Ther. 2016 Sep 27;24(11):1974–86. doi: 10.1038/mt.2016.158 (PMC5154475; doi:10.1038/mt.2016.158)
Supplement: Supplementary Figures [file mt2016158x1.doc]

**Dual Therapeutic Action of a Neutralizing Anti-FGF2 Aptamer in Bone Disease and Bone Cancer Pain**

Ling Jin, Yosuke Nonaka, Shin Miyakawa, Masatoshi Fujiwara and

Yoshikazu Nakamura

**SUPPLEMENTARY MATERIAL**


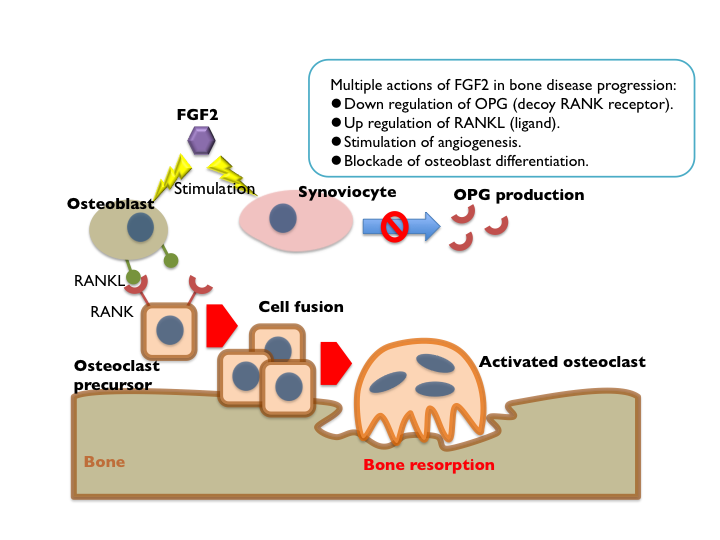


**Figure S1.** A schematic illustration of the action of FGF2 in bone disease progression.


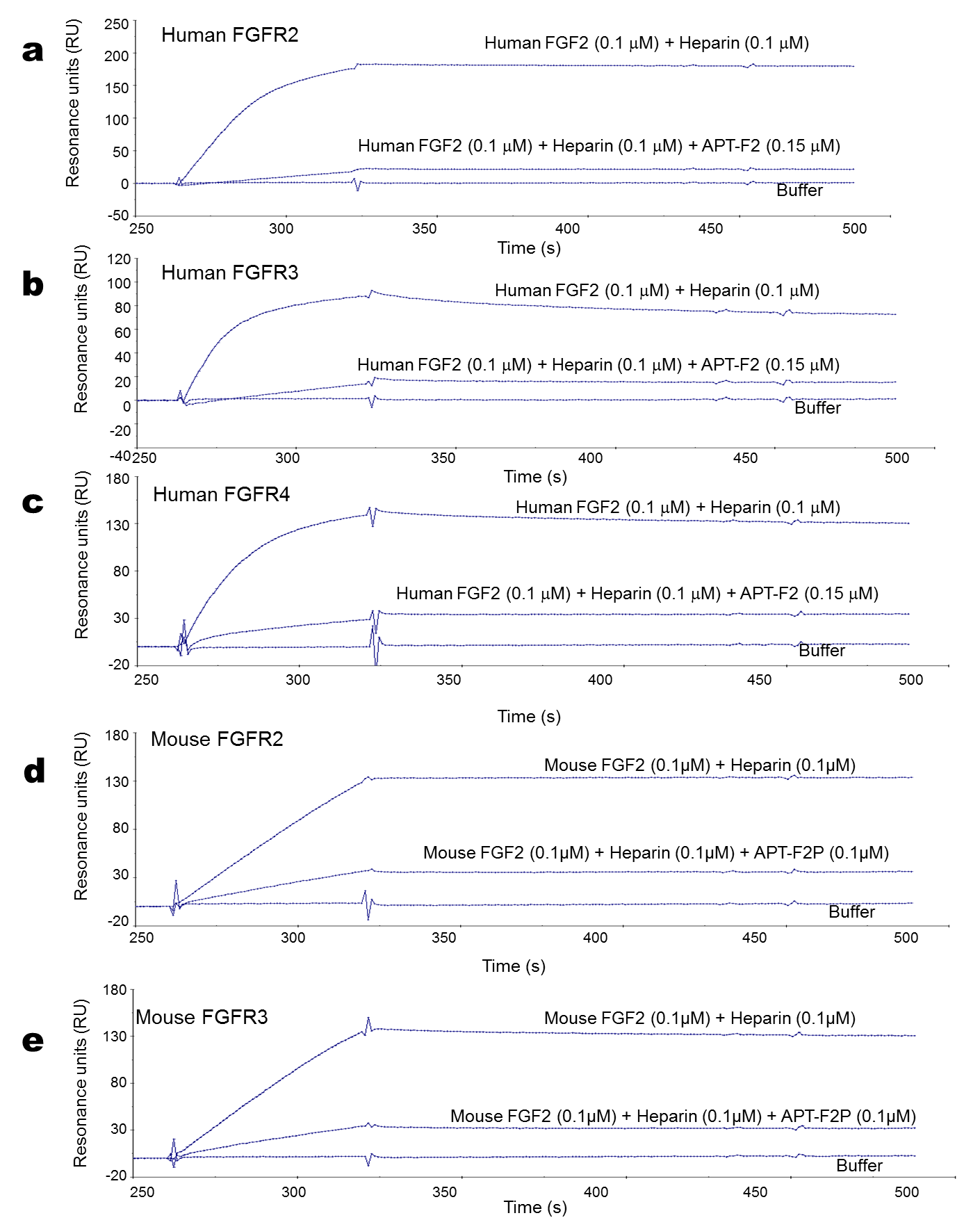


**Figure S2.** SPR sensorgrams showing the ability of APT-F2 to block the interaction of human FGF2 with human FGFR2 (**a**), human FGFR3 (**b**), and human FGFR4 (**c**). The blocked interaction of mouse FGF2 with mouse FGFR2 (**d**) and mouse FGFR3 (**e**) is also presented. Experimental conditions and procedures are as described in Fig. 1.


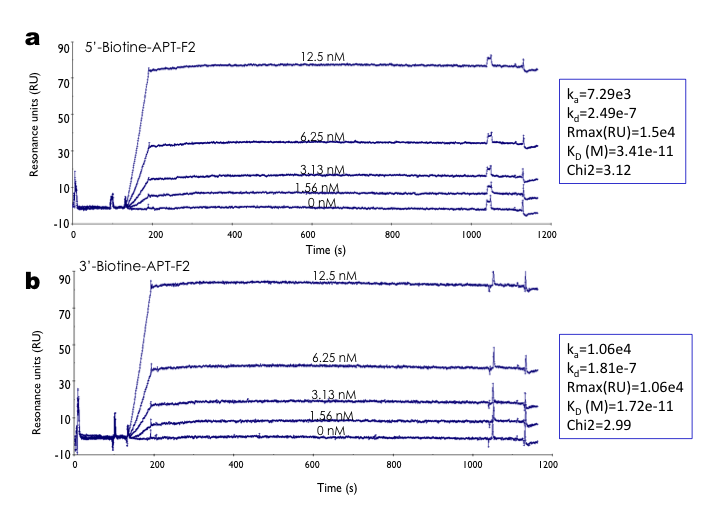


**Figure S3.** Binding profile of APT-F2 measured by SPR. Sensorgrams of APT-F2 binding to human FGF2 in different concentrations (12.5, 6.25, 3.13, and 1.56 nM from upper to lower sensorgrams) and the estimated parameters including k_a_ (association rate), k_d_ (dissociation rate) and K_D_ (dissociation constant). (**a**) 5’-biotine-APT-F2 was immobilized to the sensor chip. (**b**) 3’-biotine-APT-F2 was immobilized to the sensor chip.

**
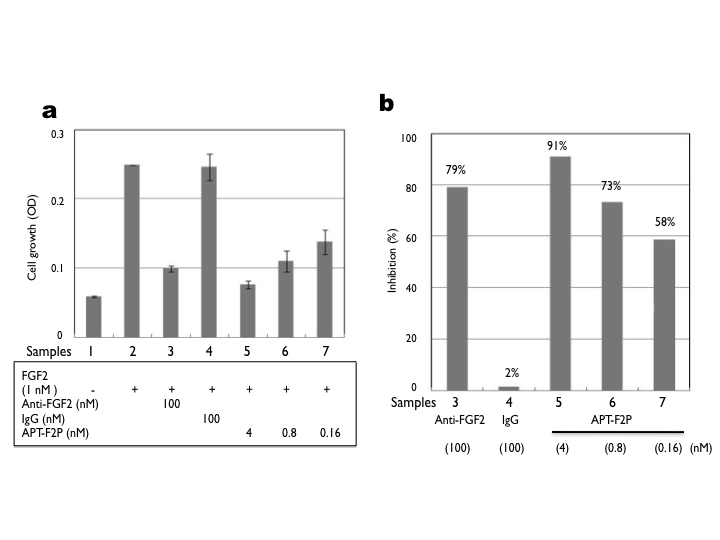
**

**Figure S4.** Attenuation of FGF2-induced proliferation of G292 cells by APT-F2P. (**a**) G292 cells growth was examined in the presence of FGF2 (1 nM) and the neutralizing anti-FGF2 antibody or APT-F2P at the indicated doses for 72 hr. (**b**) Inhibition profile of APT-F2P.


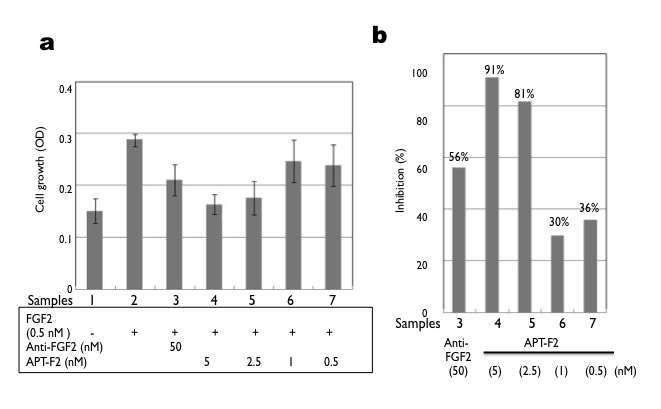


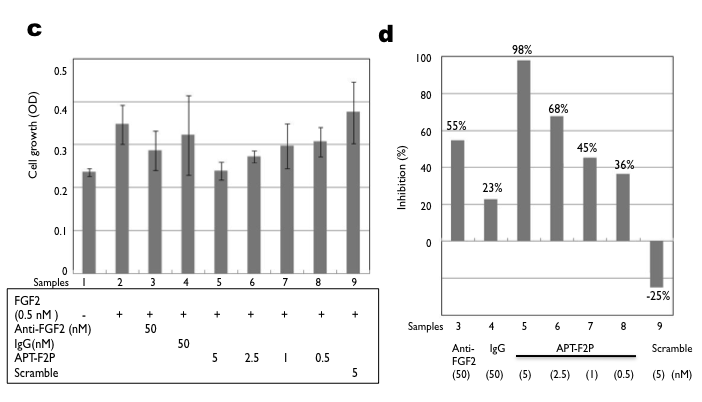


**Figure S5.** APT-F2P mediated attenuation of FGF2-induced proliferation of HFLS cells derived from two RA patients. (**a**) HFLS cells derived from RA patient (lot no. 2008 purchased from Cell Applications) were grown in the presence of FGF2 (0.5 nM) and the neutralizing anti-FGF2 mAb or APT-F2 at the indicated doses for 72 hr. (**b**) Inhibition profile of APT-F2. (**c**) HFLS cells derived from RA patient (lot no. 1462 purchased from Cell Applications) were grown in the presence of FGF2 (0.5 nM) and the neutralizing anti-FGF2 mAb or APT-F2P at the indicated doses for 72 hr. (**d**) Inhibition profile of APT-F2P.


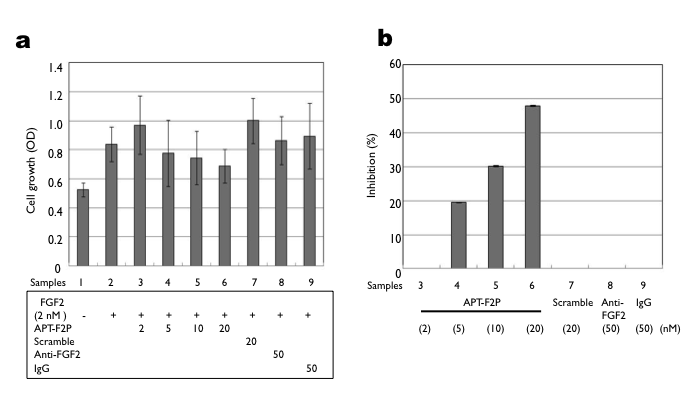


**Figure S6**. Attenuation of FGF2-induced proliferation of premature osteoblasts by APT-F2P. (**a**) Growth of MC3T3-E1 cells was examined in the presence of FGF2 (2 nM) and the neutralizing anti-FGF2 antibody or APT-F2P at the indicated doses for 72 hr. (**b**) % Inhibition of APT-F2P in the FGF2-induced osteoblast precursor cell proliferation arrest.

**
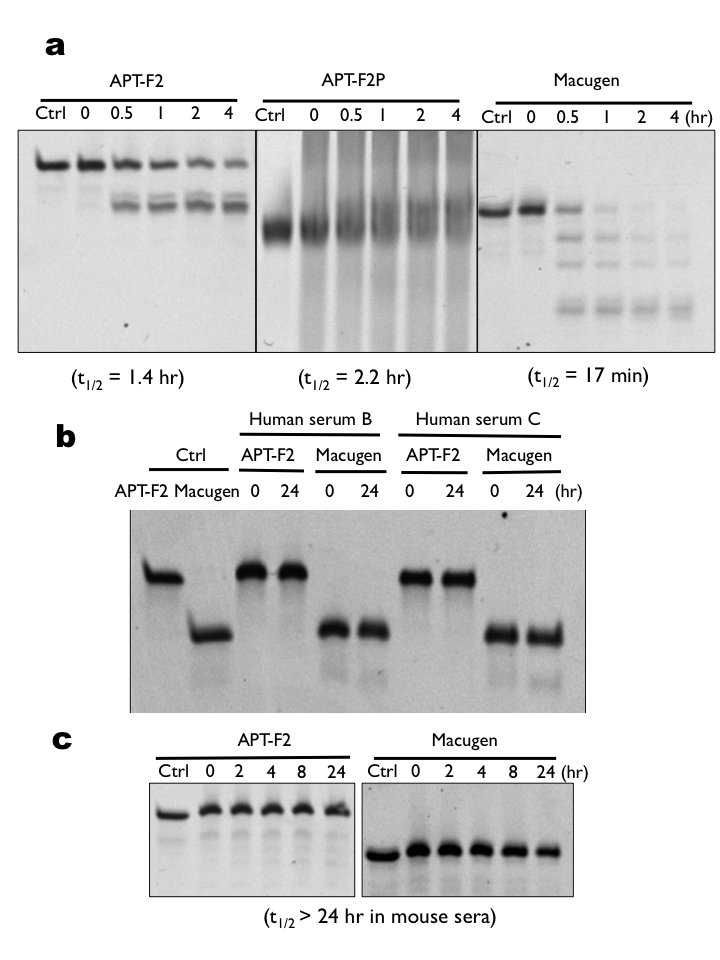
**

**Figure S7.** Stability of APT-F2 and APT-F2P in different kinds of sera. Ribonuclease activity differs among independent human sera. The most reactive human serum (designated A) was used in (**a**) to examine the distinct stability of each aptamers, and normal, less reactive, human sera (designated B and C) were used in (**b).** (**a**) APT-F2, APT-F2P and the non-PEGylated oligonucleotide of Macugen were incubated with the most reactive human sera A at 37°C for the indicated period, and analyzed by the denaturing polyacrylamide gel electrophoresis. The estimated t_1/2_ values are noted. (**b**) APT-F2 and the non-PEGylated oligonucleotide of Macugen were incubated with human sera B and C at 37°C for the indicated period, and analyzed by the denaturing polyacrylamide gel electrophoresis. (**c**) APT-F2 and the non-PEGylated oligonucleotide of Macugen were incubated with mouse sera at 37°C for the indicated period, and analyzed by the denaturing polyacrylamide gel electrophoresis. The estimated t_1/2_ value is noted.


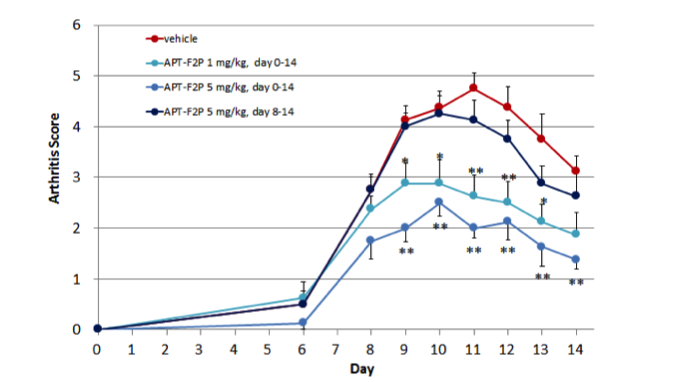


**Figure S8**. Development of GPI-induced arthritis is suppressed by APT-F2P in mice. The values of arthritis score are the mean and SEM of 8 mice per group. *P<0.05 and **P<0.01, versus vehicle at each time point (Dunnett test).


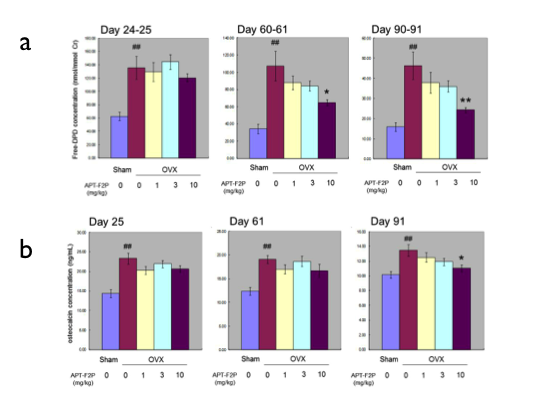


**Figure S9**. Inhibitory effects of APT-F2P on secretion of urinary DPD (**a**) and serum osteocalcin (**b**). Values are the mean and SEM of 8 mice per group. ## p<0.01 sham vs OVX groups (Student T test). * P<0.05, ** p<0.01 OVX vs APT-F2P treatment groups (Dunnett test).


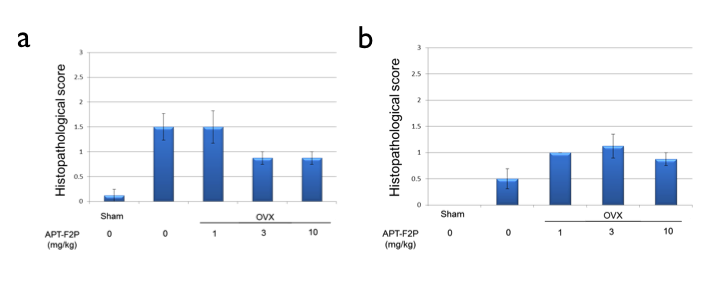


**Figure S10**. Cell numbers of osteoclasts (**a**) and osteoblasts (**b**) in femoral bones of OVX rats untreated and treated with APT-F2. Histopathological scores are the mean and SEM of 8 mice per group.


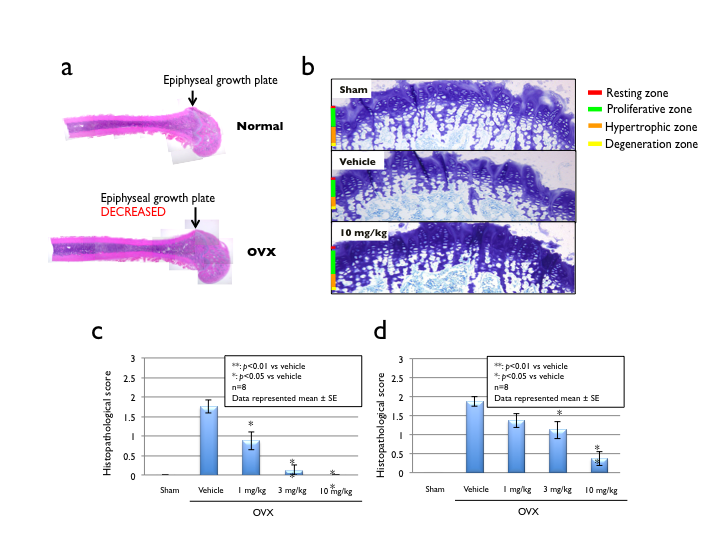


**Figure S11.** Bone histology in OVX rats. Analytical procedures are described in Methods. (**a**) Hematoxylin and eosin staining of femur. (**b**) Toluidine blue staining of epiphyseal growth plate. (**c**) Blockade of epiphyseal growth plate loss by APT-F2P. (**d**) Restoration of toluidine-blue positive area in the metaphysis by APT-F2P. Histopathological scores are the mean and SEM of 8 mice per group. * P<0.05 and ** P<0.01, versus vehicle (Steel test (**c**), and Dunnett test for (**d**)).


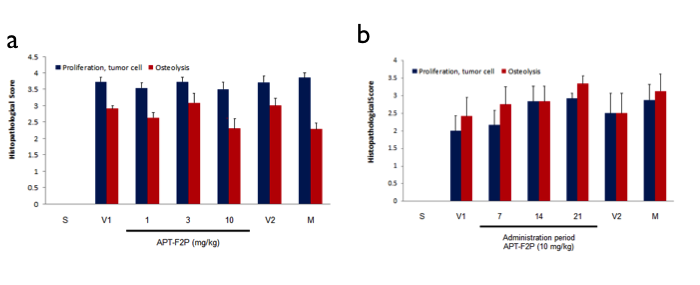


**Figure S12**. Histopathological assessment of tumor proliferation and osteolysis in Exp. 1 (**a**) and Exp. 2 (**b**) of FBC pain model as described in Figure 8. Histopathological scores are the mean and SEM of 8 mice per group.


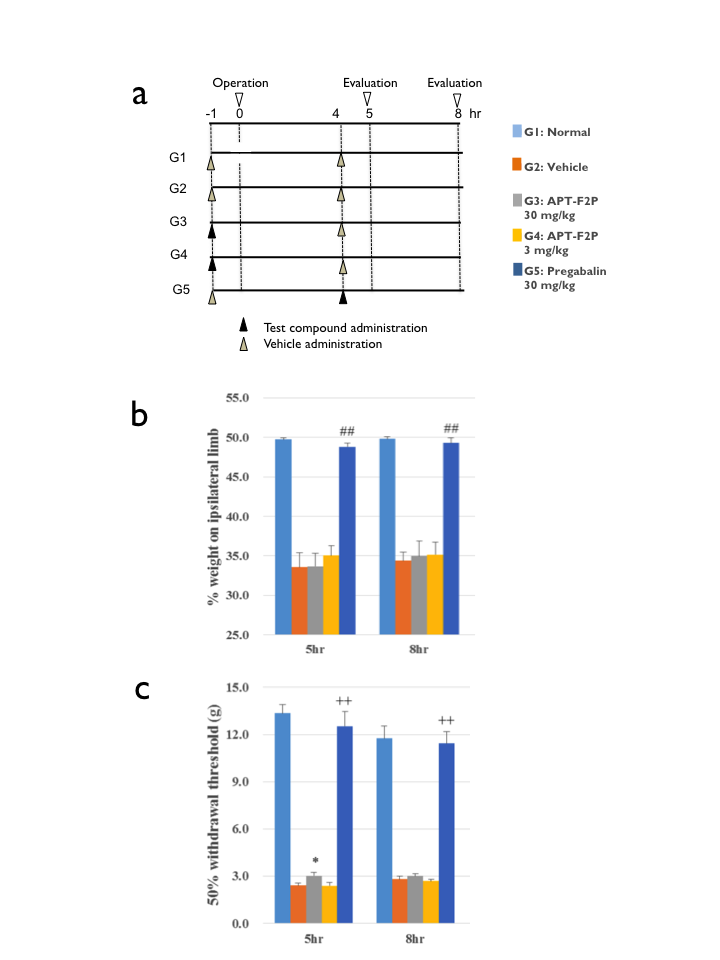


**Figure S13.** Effects of APT-F2P on a rat model of postoperative pain. (**a**) Procedures of rat postoperative pain experiments. (**b**) Weight-bearing test. (**c**) von Frey filament test. Values are the mean and SEM of 10 mice per group. ## P<0.01 versus G2 (Wilcoxon test). *P<0.05 versus G2 (Steel test).
